# A whole of school intervention for personality disorder and self-harm in youth: a pilot study of changes in teachers’ attitudes, knowledge and skills

**DOI:** 10.1186/s40479-018-0094-8

**Published:** 2018-10-02

**Authors:** Michelle L. Townsend, Annaleise S. Gray, Tanya M. Lancaster, Brin F. S. Grenyer

**Affiliations:** 1 School of Psychology and Illawarra Health and Medical Research Institute, University of Wollongong, Wollongong, NSW Australia; 2 Department of Education, Sydney, NSW Australia

**Keywords:** Schools, Mental health, Teachers, Self-harm, Personality disorder, Training

## Abstract

**Background:**

The school environment offers an ideal opportunity for early identification and intervention for youth with self-harm and complex mental health issues, such as borderline personality disorder (BPD). Yet, class teachers often report minimal knowledge, feeling ill-equipped to respond, and experience high levels of stress when exposed to such challenges. Research is required to understand how training and development activities led by school counsellors may enhance teacher attitudes, confidence and knowledge of self-harm and complex mental health issues, such as BPD. We aimed to explore the extent that a whole of school initiative might enhance capacity for early identification and intervention.

**Method:**

18 secondary schools implemented a manualised program, Project Air Strategy for Schools. *N* = 400 class teachers (71.3% female, mean age 42 years) across city and rural locations were evaluated before and after program implementation on attitudes, knowledge and skills.

**Results:**

Providing class teachers with additional training on complex mental health issues and associated behaviours such as self-harm was well received. Participants reported post-program improvements in their optimism (*d* = .35), confidence (*d* = .63), knowledge (*d* = .73) and skills (*d* = 0.67) in working with young people with complex mental health issues, such as BPD.

**Conclusions:**

Providing school counsellor led structured approaches, to help class teachers identify and respond to youth in distress, closed identified gaps. Results indicated improvements in class teachers’ knowledge and attitudes towards self-harm and BPD. The intervention also improved the capacity of schools to plan and implement strategies to reduce the impact of mental health problems on the young person and their peers. A stay-at-school psychological care approach was fostered by enhancing partnerships between class teachers and school counsellors.

## Background

Personality disorders are of high prevalence in the global population, with estimates at 6.1% [1]. Personality disorders typically emerge in adolescence through a combination of genetic vulnerability and environmental triggers [2–4]. Biological factors are thought to interact with social environment factors including dysfunctional or invalidating and traumatic early attachment or maltreatment [3, 5]. Childhood trauma has a central role in the development of mental disorders [6] and childhood adversity predicts a range of psychological difficulties that can prevail across the lifespan [7, 8]. Personality disorders are considered a continuation of precursor features as well as maladaptive traits that first emerge during childhood or early adolescence [9–11]. These include substance use disorders, disruptive behaviours, and difficulties with attention and emotion regulation [12]. Borderline personality disorder (BPD) can therefore exacerbate many of the developmental tasks of adolescents. The process of identity formation that coalesces in adolescence is likely to be especially challenging for those at risk, as they attempt to integrate different representations of self-other relatedness into a coherent whole [13].

Children and adolescents with emerging BPD features are likely to experience clinical distress and psychosocial difficulties which can first emerge during the schooling years [14]. Young people exhibiting subthreshold BPD features, experience more severe mental illness and exhibit poorer social and occupational functioning compared to young people not exhibiting BPD features [15]. If untreated, these children and adolescents are at risk of experiencing significant social, educational, employment and financial impairments later in life [16, 17]. Despite the longstanding reluctance to diagnose young people with BPD [3, 18], due to perceptions of poor prognosis and lack of effective treatments [19], a diagnosis can be made under 18 years of age [20] and assessment of BPD features in adolescents and subsequent intervention is recommended by the National Health Medical Research Council (NHMRC) [21]. Further, early recognition of these features in at-risk children and evidence based intervention has the potential to alter the trajectory of the disorder [14]. Early interventions may require less intensity and be more effective than waiting until adolescence or later (e.g. [22]). However, there is a need to ensure that children and young people are assessed for BPD using a dimensional and categorical approach to ensure interpersonal functioning is fully understood across multiple domains [23].

Self-harm is a leading public health issue concerning adolescents internationally, with a lifetime prevalence estimated at 16.1–18% in adolescents aged 11–18 years [24]. Research in the United Kingdom has identified that the rates of young women aged between 13 and 16 years engaging in self-harm have increased by 68% in the period 2011–14 [25]. Over the last decade self-harm behaviours have been shown more prominently in the media, music, videos, and magazines [26]. Although self-harm is not specific to BPD and is prevalent in a range of mental health illnesses, recent research has indicated that among a BPD sample, self-harm is a key predictor of mental health care utilisation and is a significantly better target symptom than an overall BPD construct [27] The causes of self-harm behaviours are complex and there are a range of theories about why young people engage in this behaviour. Some self-harm is one-off and experimental [28], while other behaviours continue in negative cycles. Overall, the evidence suggests the function of self-harm is largely affect regulation [29], but it is likely that self-harm serves several functions concurrently [30]. Self-reports from young people offer a range of reasons such as attempting to control, escape from, or avoid difficult and overwhelming feelings and emotional pain, expressing anger, feeling ‘something’ (e.g., if feeling numb or dissociated), self-punishment, or communicating a need for help [31, 32]. Self-harm and impulsivity, along with feelings of emptiness worsen prognoses after 12 months [33].

The challenges of responding to this issue are apparent in all areas of the schooling and health systems. Schools can struggle to identify and respond and education staff can be overwhelmed and confronted by self-harm behaviours [34, 35]. In the health system, from general practice to emergency departments in hospitals, practitioners anecdotally report seeing an increase of young people presenting with self-harm injuries. The cost of self-harm treatment in hospital for young people aged 16 and under in Australia over a 10 year period has recently been estimated at AUD$64 million (USD$48 million) [36]. Hospitalisations for self-harm are increasing for males, however, females aged 11–16 years continue to have a higher hospitalisation rate at 5:1 [36]. More than half of all self-harm incidents requiring hospitalisation occurred at home, being 59.7% of 6–10 year olds and 54.8% of 11–16 year olds. School was the place of occurrence for 8.1% of children ages 6–10 years and 5.7% of 11–16 year olds [36].

As the setting where young people spend a significant proportion of their childhood and adolescence, schools are at the forefront of responding to student self-harm and mental health. Over the last 30 years a whole of school approach developed by the World Health Organisation (WHO) has been implemented across the globe to promote the health and well-being of students. Key components of this approach include: the educational curriculum, the social and physical environment, the policies and practices of the school, school health services, and school, home and community engagement [37]. Promoting social and emotional wellbeing for students has been linked to better outcomes educationally [38, 39] and promotes student mental health [40–42].

As there has been limited research on students with personality disorder in the school setting, more research is needed into programs such as the WHO whole of school approach. The studies to date indicate that the school environment exerts both positive and negative influences on personality disorder features [43, 44]. Schools with a high focus on learning were associated with declines in Cluster B (Borderline, Narcissistic, Histrionic, Antisocial) features and schools high in opportunities for student autonomy were associated with declines in Cluster A (Paranoid, Schizoid, Schizotypal) features [43]. Whereas schools with conflictual environments or informality between students and teachers were related to increases in Cluster A and C (Avoidant, Dependent, Obsessive-Compulsive) features [43]. Hengartner MP, Ajdacic-Gross V, Rodgers S, Müller M and Rössler W [44] found adversity experienced in the school particularly through bullying victimisation and conduct problems was associated with all personality disorders. Therefore, the evidence to date suggests negative experiences in the school environment appear to have a sizeable impact on personality disorder symptomology.

Class teachers can experience difficulties understanding young people with personality disorder and can be challenged by the behaviours in the school environment. Teachers may resort to disciplinary approaches to address the students’ expressions of anger, impulsivity and reactivity of mood. Difficulties in relationships with peers and adults in the school are also likely, along with other risky behaviours including self-harm, suicidal ideation, sexual behaviours, violence and criminal activity [45]. This constellation of presenting issues may result in students being suspended and expelled from school. For young people with complex mental health issues the risks of leaving school early, without necessary qualifications are high and have a profound impact on their lifetime legacy [46]. It is important to differentiate between young people with emerging conduct disorder and callous unemotional traits compared to those with emotional hypersensitivity and attachment neediness. Schools and young people will benefit from clear attention to the function of behaviours in understanding the best response [47].

Schools are increasingly being recognised as important sites to respond to student mental health concerns [48]. The early recognition by class teachers of these student mental health issues can facilitate stronger links to school counsellors that use evidence-based approaches. Education staff can also work collaboratively with parents or caregivers to support the young person where they may have an identified pastoral role. This work can be challenging to educational staff who may require additional knowledge and skills to create an environment conducive to promoting the mental health of students [49]. Professional development and collegial support is likely to be key to supporting schools in this work [41]. However, teachers report they receive little training and pre-service education to support them in this role [50]. Over recent years the training available to teachers in regards to student mental health is slowly increasing, but knowing which training is reputable and evidence based can also be difficult to determine Furthermore, while there are a number of training programs in existence that focus on self-harm and mental health, none to our knowledge have a specific focus on personality disorders.

To address these issues, we developed a whole of school framework and provided guidelines, accredited training, resources and opportunities to collaborate together as educational professionals, targeted at enhancing their identification, understanding and capacity to respond to complex mental health issues, such as personality disorder. The aim of this study was to evaluate the extent that participating in accredited training provided by school counsellors increases the ability and confidence of class teachers to respond to students with complex mental health presentations and self-harm in the school setting.

## Method

### Participants

Participants were school teachers (*N* = 400) from 18 public schools in New South Wales, Australia. Participants were predominantly female (*n* = 285, 71.3%), average age 42.02 years (*SD* = 11.42, range 19–69). The schools undertaking the program were from rural and remote areas (*n* = 10), while the remaining were closer to major cities. Participants provided consent to be part of the evaluation of the program and voluntarily completed pre and post measures.

### Intervention

Project Air Strategy is a Personality Disorders Strategy that aims to enhance treatment options for people with personality disorder and their families and carers. The strategy endorses an integrative collaborative relational approach and works with health services, agencies, clinicians, families and carers, and consumers, to improve treatments for personality disorder [51]. Project Air Strategy for Schools supports secondary schools to enhance their understanding and responses when working with young people with complex mental health issues and presentations, including personality disorder, trauma history, self-harm and suicidal behaviour and difficulties with identity, emotions and relationships. The program aims to support all professionals working with young people including school counsellors, health staff, welfare workers, teachers and school administrators. Guidelines have been developed, using a whole of school approach, to inform actions by education staff when supporting young people with complex mental health presentations [47]. These guidelines are then used in individual schools to examine current policies and practices in supporting students. The program and resources as part of Project Air Strategy for Schools focuses on five key areas for schools supporting young people with complex mental health issues, and each area utilises evidence based strategies, information and resources, outlined in Table 1.

**Table 1:** Focus Areas of Project Air Strategy Intervention

1. Understanding Complex Mental Health Problems; - Develop an understanding of personality disorders, its prevalence, symptoms, and risks - Develop an appreciation for the experience of a young person with emerging personality disorder - Develop insight into the role of compassionate communication - Understand issues of trauma, self-harm and suicide ᅟ 2. Identifying and Assessing Risk; Responding to Crisis and Self-Harm Situations; and Responding Effectively to Challenging Behaviourss; - Develop an understanding of self-harm and its function within the context of a mental health disorder - Develop an understanding of how to manage risks of ‘social contagion’ between young people - Responding to challenging behaviours and incidents - Develop an awareness of how personal reactions can escalate or de-escalate student behaviour - An understanding of the different roles of teachers, welfare team, school counselling staff and health workers in supporting intervention and treatment ᅟ 3. Working to Improve the School Environment; - Whole of school planning to support student wellbeing - Implementing reasonable adjustments to ensure young people with a personality disorder are encouraged to attend and complete school - Identifying learning and support strategies that foster student engagement - Utilising individualised care planning process to support students 4. Teacher Wellbeing; - Develop an understanding of the importance of self-care and teacher wellbeing - Utilising peer debriefing and consultations with school counsellors ᅟ 5. Working With Parents with a Personality Disorder. - Establish and maintain respectful, collaborative relationships with parents/carers regarding their child’s learning and wellbeing	

**Fig. 1:**
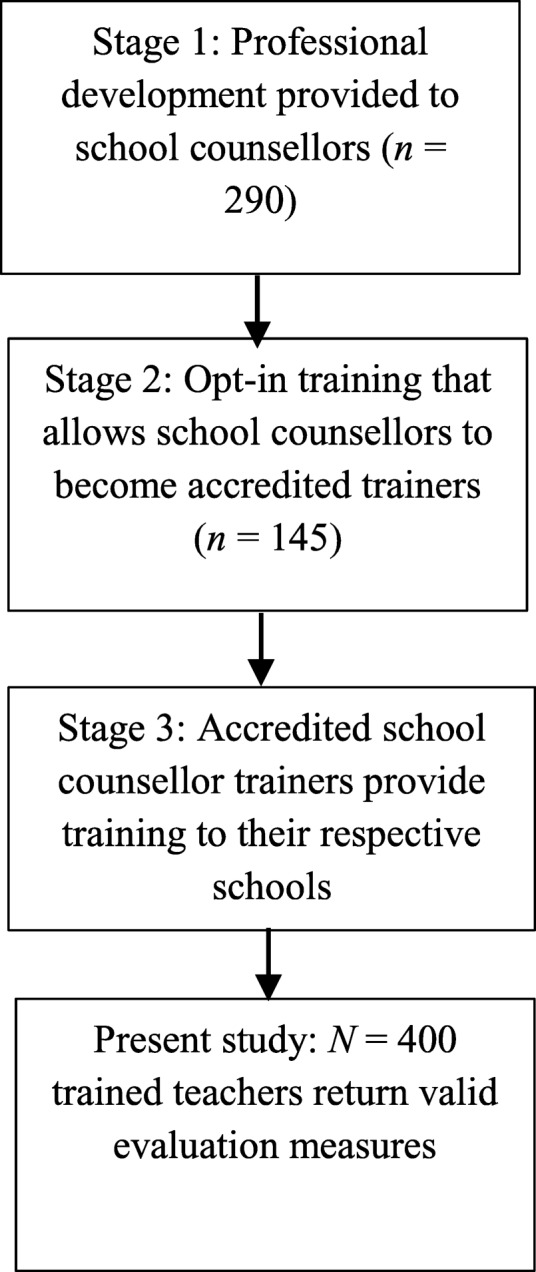
Flowchart of intervention program delivery

The program consisted of three components (see Fig. 1). The first component was a professional development day provided to school counsellors and psychologists from across the state (*n* = 290). This workshop provided an update on personality disorder theory, research, and evidence-based practice and skills-training for identifying and responding to young people with complex mental health needs. Following this training day, participants opted to attend the second component, which would accredit them to implement the final component, accredited training to education staff in their local schools. The full-day consultation workshop explored issues of complexity in school students and optimal ways to working with school education staff. Attendees were given training and practice in delivering the workshop in their local schools. Accredited trainers facilitated the final component, being the delivery of an accredited workshop in local schools to up-skill school teachers to identify, understand and respond to students with complex mental health problems, including self-harm, suicide, trauma and emerging personality disorder.

This study reports the results of this final component of the train-the-trainer model. In this professional development, school counsellors worked with class teachers to implement a whole of school approach to create positive learning environments and promote student wellbeing. The course developed strategies to successfully support and engage students who are experiencing complex mental health problems and challenging behaviours. The program was operationalised into two modules. Module one focused on personality disorder (PD) prevalence, features and risks, common experiences of young people with PD, practical opportunities for fostering relationships, how to put a care plan in place to support young people with PD and the role of other stakeholders in supporting students. Module two focused on self-harm and its functions; appropriate responses to self-harm and suicidal ideation, understanding and managing contagion and self-care for education staff.

### Measures

#### Knowledge of deliberate self-harm questionnaire (KDS)

The KDS was developed by Crawford T, Geraghty W, Street K and Simonoff E [52] to assess health professionals knowledge of deliberate self-harm in adolescents. A revised 10-item measure has been used in studies of similar education cohorts (see [53]) and in the present study. A single measure of knowledge is obtained by summing the total number of correct true/false responses, with a possible maximum score of 10. For additional insight in to teacher’s knowledge of self-harm, participants were asked to estimate the prevalence of self-harm and the age of onset of self-harm.

#### Attitudes towards deliberate self-harm questionnaire (ADSHQ)

The ADSHQ was originally developed by McAllister M, Creedy D, Moyle M and Farrugia C [54] to explore emergency department nurse’s attitudes and response to deliberate self-harm. The questionnaire was later revised and validated by Berger E, Hasking P and Reupert A [55] for use on education staff. 11-items were included and responses are made using a four-point Likert scale from 1 (strongly disagree) to 4 (strongly agree). Higher scores indicate more positive attitudes towards deliberate self-harm. Reliability analysis indicated acceptable internal consistency (factor 1 dealing effectively with self-injuring students *α* = .83 pre, *α* = .89 post; factor 2 perceived confidence in assessment and referral of self-injury students *α* = .68 pre, *α* = .79 post). The present study did not include items loading on factor 3 ‘ability to cope effectively with legal and school regulations that guide practice’ as these items appeared to conflict with the current training content.

#### Attitudes and skills questionnaire (ASQ)

The ASQ [56] is 6-item scale originally developed to measure clinicians’ ability and desire to work with clients with BPD. This study revised the measure, asking participants to rate their optimism, confidence, knowledge and skills in working with young people with complex mental health issues and young people who self-harm respectively, on a rating scale from 1 (low) to 5 (high). Higher scores indicate more positive attitudes.

#### Expertise rating

To understand their baseline expertise, participants were asked to rate their expertise in working with young people who have complex mental health issues as minimal, developing, sound, advanced or expert. Participants were also asked to estimate the percentage of high school students that self-harm, the typical age of onset of self-harm behaviour, and whether self-harm behaviour is on the increase on a 5-point likert scale (strongly agree, agree, neutral, disagree, strongly disagree).

#### Program evaluation

Participants were asked to rate how satisfied they were with the program on a 4-point likert scale (not satisfied, somewhat satisfied, satisfied, very satisfied). Participants were also asked to rate how helpful they believe the program is in improving outcomes for young people with complex mental health issues and how helpful they found the program resources (e.g. film, guide, factsheets) on a 4-point likert scale (not helpful, somewhat helpful, helpful, very helpful) respectively. Participants were also asked whether they would recommend the program to a colleague, rated on a binary yes/no variable.

### Data analysis strategy

We were interested in the impact of the program to change knowledge, attitudes, and skills to intervene with young people. Descriptive statistics described participants’ current level of expertise prior to participating in the program while a general linear model evaluated changes in participant’s knowledge, attitudes and skills in working with complex mental health issues such as BPD and self-harm. Qualitative data was collected from participant’s responses to the question ‘what aspects of this training did you get the most out of?’ Data was analysed in NVivo version 11 [57] to generate codes and subsequent themes supported by the data. Each theme was interrogated to determine the most appropriate quotes to use. Their formulated meanings were interpreted by one researcher (ASG), and confirmed by the remaining two lead researchers (MLT, BFSG).

## Results

### Participant characteristics

Most teachers rated their expertise in working with young people who have complex mental health issues (for example, personality disorder, trauma history, self-harm and suicidal behaviour) as minimal (*n* = 61, 15.5%) or developing (*n* = 158, 39.5%). Others self-identified as being expert (*n* = 1), advanced (*n* = 28, 7.0%) or sound (*n* = 132, 33.0%). Nineteen teachers (4.8%) did not indicate their level of expertise.

Responding teachers (*n* = 381) estimated that on average 20.38% (*SD* = 16.51, range 1–90) of high school students self-harm. The majority (*n* = 249, 62.7%) of responding teachers (*n* = 397) indicated that they agreed that self-harm behaviour is on the increase, with a further 18.10% (*n* = 72) strongly agreeing. Responding teachers (*n* = 386) estimated that the mean typical age of onset for self-harm behaviour is 12.46 year (*SD* = 1.92, range 3–19).

### Program evaluation

The majority (*n* = 258, 87.8%) of responding teachers (*n* = 294) reported being satisfied or very satisfied with the program. Most responding teachers (*n* = 295) also indicated that the program was helpful (*n* = 130, 44.1%) or very helpful (*n* = 115, 39.0%) in helping them to work with the student, school counsellor and school environment to improve outcomes for young people with complex mental health issues. 91.7% (*n* = 266) of responding teachers (*n* = 290) indicated that they would recommend the program to a colleague. Regarding the helpfulness of the training’s associated resources, the majority (*n* = 231, 79.4%) of responding teachers (*n* = 291) indicated that the resources were helpful or very helpful.

Participants also had the option to report what they got the most out of the program. Three themes were identified by teachers: 1) validation of current support that teachers provide to young people with complex mental health issues; 2) increased knowledge, understanding and skills to intervene with young people with complex mental health issues; 3) promotion of a whole of school approach and collegial discussion. Table 2 presents significant statements and their formulated meanings.

**Table 2:** Significant statements and their formulated meanings of class teachers’ qualitative responses to the implementation of the Project Air Strategy Schools intervention program

Significant statements	Formulated meanings
a. Whole staff discussion about issues specifically relevant to [school name].	a. The program facilitated open discussion about complex mental health issues in their respective schools.
b. More equipped to “be there” if a student needs help.	b. The program increased teachers’ skills and confidence to support students with complex mental health issues.
c. The importance of sharing the load: a team approach.	c. The program promoted a whole of school approach.
d. Reassured [that we are] already doing lots of positive actions that will help students.	d. The program reinforced the support teachers are already providing.
e. Learning about what personality disorders are and the procedures and policies for dealing with students at risk of self-harm or suicide.	e. The program increased awareness and knowledge about personality disorder.
f. Talking with other colleagues about self-harm and suicide.	f. The program promoted collegial discussion about self-harm and suicide.
g. Ensuring teachers understand that they are not diagnosing – they are responding to a behaviour.	g. The program provided role clarification to teachers regarding personality disorder.
h. Information on how language that is commonly used by teachers can create stigma for young people with complex mental health issues. Importance of treating behaviour rather than waiting for [a] formal diagnosis.	h. The program provided knowledge about personality disorders.

### Participant knowledge, attitudes and skills

Results indicated improvement in knowledge regarding deliberate self-harm (Table 3). Results showed an increase in positive attitudes towards young people engaging in deliberate self-harm following program delivery, as indicated by a statistically significant (*p* < .001) difference on total ADSHQ score (Table 3). Items loading on factor 1 indicated a statistically significant (*p* < .001) improvement in teacher attitudes, while items loading on factor 2 did not significantly differ post program (*p* = .225).

**Table 3:** Pre and post mean and standard deviations for paired samples t-test

	Pre	Post	*t*	*df*	95% CI	*d*
KDS	6.79(1.58)	7.20(1.48)	−3.770*	293	[− 0.62, − 0.19]	0.27
ADSHQ total	32.77(4.05)	35.51(4.01)	−9.942*	154	[− 3.29, − 2.20]	0.55
Factor 1^a^	11.91(2.96)	14.33(2.62)	− 12.863*	171	[− 2.79, − 2.05]	0.69
Factor 2^b^	21.07(2.09)	21.24(2.24)	−1.217	241	[−0.45, 0.11]	
Self-harm						
Optimism	3.19(1.06)	3.74(0.90)	−11.417*	289	[− 0.65, − 0.46]	0.44
Confidence	2.80(1.04)	3.60(0.85)	−16.084*	293	[− 0.90, − 0.70]	0.67
Knowledge	2.78(0.95)	3.71(0.81)	−18.308*	292	[− 1.02, − 0.83]	0.84
Skills	2.53(0.96)	3.44(0.87)	−18.114*	288	[− 1.01, − 0.81]	0.80
Complex mental health issues						
Optimism	3.20(1.07)	3.64(0.94)	−8.930*	290	[− 0.53, − 0.34]	0.35
Confidence	2.74(0.99)	3.48(0.90)	−15.252*	291	[− 0.84, − 0.65]	0.63
Knowledge	2.72(0.99)	3.56(0.83)	−16.400*	291	[− 0.95, − 0.74]	0.73
Skills	2.58(0.97)	3.34(0.90)	−16.268*	292	[− 0.84, − 0.67]	0.67

*KDS* Knowledge of Deliberate Self-Harm Questionnaire, *ADSHQ* Attitudes Towards Deliberate Self-Harm Questionnaire

The sample size for different measures varies due to missing values and availability of post data

*p < .001

^a^Dealing effectively with self-injuring students

^b^Perceived confidence in assessment and referral of self-injuring students

Following program delivery, results on the revised ASQ items provided further support for participants’ improvement in their optimism, confidence, knowledge and skills in working with self-harm (*p* < .001). Moreover, improvement in teachers’ optimism, confidence, knowledge and skills also improved in relation to complex mental health issues (*p* < .001) (Table 3).

## Discussion

This pilot study described here was aimed at investigating changes in teacher’s attitudes, knowledge and skills following the implementation of a new whole of school model for helping young people with complex mental health issues. Results indicated that teachers’ knowledge of self-harm improved following the program. Further, that their attitudes toward young people engaging in deliberate self-harm were significantly more positive following the program. Regarding complex mental health issues such as personality disorder, this study indicated that teachers improved in their optimism, confidence, knowledge and skills in working with this population after completing the program. These findings support the value of early intervention in the school setting, and reinforces the idea that with appropriate support and training, teachers can assist in the identification and referral of young people with complex mental health issues like personality disorder. Qualitative findings indicate the strength of improving the school counsellor - class teacher relationship to foster early identification, support and intervention for youth. Thus the stay-at-school psychological care approach provided appeared to foster partnerships between class teachers and school counsellors to work with identified students within the school setting.

Interestingly, ADSHQ factor two items, perceived confidence in assessment and referral of self-injuring students, did not significantly differ following the program. However, items loading on factor one, dealing effectively with self-injuring students, significantly improved. This result may also reflect the training content, which primarily targeted the effectiveness of education staff in working directly with this population, rather than their ability to assess and refer.

There are a number of strengths in this study. The whole of school approach engaged multiple levels of education staff and fostered collaborative practice. Teachers reported receiving high quality accredited training in the complex mental health needs of students particularly personality disorders. The findings reported reinforce that professional development and opportunities for education and counselling staff to meet together and focus on their specific school environment is effective. Through this initiative local schools were able to consider their own context regarding student well-being, responses to crisis situations, management of challenging behaviours as well as ongoing school, classroom and social environment improvements. This framework is likely to represent a cost effective approach that benefits all students as well as students with complex mental health issues [43, 58]. Longer-term savings are also likely to be produced if evidence based treatment is provided at crucial point in time [59], potentially changing the trajectory of young people with a personality disorder.

The model of providing professional development to local school counsellors, who then were trained in delivering the accredited training in their schools, ensured that trainers provided context for the program implementation e.g. if the school had experienced the recent suicide death of a student, this could be taken into account when delivering the program. The study also provides support for the cascade or train-the-trainer model [60] of delivery for these types of initiatives, which is likely to be a cost effective way of training school communities. Similarly, this model of training allows for the provision of training to rural or remote areas, who may not otherwise have had access. We found that this delivery approach enhanced the relationship, information sharing and referrals between teachers and school counsellors.

There are a number of limitations to this study. First, we only had teacher feedback and thus do not have other measures of implementation (e.g. student outcomes) within the broader school context. Furthermore, schools had flexibility in the way they offered the program, in that some schools completed the training in a 4 h block, others had week or longer delay between the first 2 h session and the second 2 h session. This may place limitations on the findings as the time between completing the program varied across the schools. However, this also highlights the advantage of the flexible timing of training, allowing trainers to present the program at a time that suits their school context. It was also difficult to discern fidelity of the facilitators to the manualised program. However all trainers were provided the same accredited train-the-trainer modules and supporting resources to present in schools.

Having undertaken this pilot study, a more comprehensive research program is required. Randomised Control Trials are the gold standard in evaluating interventions, despite their limitations in the education setting [61]. Also needed are effectiveness studies, and studies of prevention and early intervention [62, 63]. Studies need to account for training and intervention fidelity, and carefully document practices that support outcomes sought [61]. There is also a need for multi method approaches that not only tests the feasibility of implementation in different schools, but also tests causal effects for educators and students following implementation. This will support an examination of whether the current study’s findings for teachers are maintained over time and an appreciation of whether changes to school climate and teacher’s knowledge, confidence and skills influence outcomes for students with complex mental health issues.

## Conclusions

Schools are important locations for addressing student wellbeing, because of the reach and familiarity to students and families, the opportunities they afford for mental health promotion and prevention as well as the link between wellbeing and learning outcomes. Teachers have established relationships with students and are ideally placed to notice changes in students’ behaviour that may indicate a mental health concern. We report that providing teachers with appropriate knowledge and skills improved their confidence in supporting and working collaboratively with school counsellors to achieve better outcomes for young people with personality disorder.
